# Numerical Simulation of Conical and Linear-Shaped Charges Using an Eulerian Elasto-Plastic Multi-Material Multi-Phase Flow Model with Detonation

**DOI:** 10.3390/ma15051700

**Published:** 2022-02-24

**Authors:** Geum-Su Yeom

**Affiliations:** Department of Mechanical Engineering, Kunsan National University, Gunsan 54150, Korea; gsyeom@kunsan.ac.kr; Tel.: +82-10-3474-5082

**Keywords:** shaped charge, elasto-plastic solid, hydrocode, multi-material multi-phase flow, detonation, Johnson–Cook model, radial return mapping algorithm, ghost fluid method, level-set method

## Abstract

This study developed a hydrocode to numerically simulate both conical and linear-shaped charges using an Eulerian multi-material and multi-phase flow model. Elasto-plastic solids and the detonation of a high explosive charge were modeled using a Johnson–Cook material model and the programmed burn model, respectively. Further, the plasticity of the solids was calculated using a radial return mapping algorithm. The model was solved using a high-resolution computational fluid dynamics (CFD) technique on Cartesian grids. Material interfaces were tracked using the level-set method, and the boundary conditions were imposed using the ghost fluid method. The developed hydrocode was validated using high-speed impact problems. Consequently, the developed hydrocode was used to successfully simulate the evolution and penetration of metal jets in shaped charges after a detonation.

## 1. Introduction

A shaped charge is a highly explosive charge for focusing explosive energy in one direction using the Monroe effect (or the Neumann effect) [1]. Generally, a shaped charge consists of a high explosive with a hollow cavity, a thin metal liner, a detonator, and a metal casing. When an explosive is ignited, a high-pressure and high-temperature explosive gas, whose pressure is concentrated in the center of the metal liner, is generated, which results in large deformation. Consequently, this results in the plastic deformation of the liner beyond its elastic limit and the formation of a hypervelocity metal jet with a typical speed and temperature of 8–12 km/s and approximately 1200 K, respectively. Shaped charges are used in numerous military and industrial applications, such as high-explosive anti-tank warheads [2], emergency escape devices for aircraft pilots, stage and fairing separation devices for space rockets [3], and for the removal and destruction of engineering facilities. Depending on their shape, shaped charges are mainly divided into conical- and linear-shaped charges, wherein the conical-shaped charge is used for penetration-related applications, and the linear-shaped charge is used for cutting objects.

Accordingly, numerous studies have investigated the types of shaped charges. Walters and Zukas [1] summarized the general theory of a shaped charge. In addition, Molinari [4] investigated the conical-shaped charge using the finite element method. Further, Liu et al. [5,6] compared two types of conical-shaped charges with conic and semi-elliptic cavities using the meshfree particle simulation. However, no liner and casting materials were added to the model. Ma et al. [7] employed a hydrocode to analyze conical-shaped charges consisting of steel and copper liners using the operator splitting method, and compared the penetration performance of the shaped charges at various liner angles. Kim and Yoh [8] presented the boundary conditions for the reactive ghost fluid method in energetic multi-material flows. Zhang et al. [9] compared a conical-shaped charge made of a copper liner to that made of a tungsten-copper liner using a commercial software (AUTODYN). Chen and Liu [10] simulated a conical-shaped charge using an elasto-plastic solid model in the Eulerian framework and solved it using the CE/SE scheme. However, because no casting material was added to the configuration, the accuracy of the computed results was limited. Sambasivan et al. [11,12] calculated the large deformation and penetration problems of high-speed elasto-plastic solids using the Convex Essentially Non-Oscillatory (CENO) scheme and the ghost fluid method. However, since their model did not consider a high explosive, it cannot simulate the shaped charge problems. Feng et al. [13] computed a linear-shaped charge using the smoothed particle hydrodynamics (SPH) technique. Recently, Yi et al. [14] analyzed a shaped charge made of a polymer liner rather than metal. Pyka et al. [15] simulated a shaped charge using a commercial software (ABAQUS), and modeled the metal jet using the smoothed particle hydrodynamics method. Du et al. [16] numerically simulated conical-shaped charges to investigate the penetration performance of a jet using a commercial software (LS-DYNA), and compared the numerical results to the experimental results. Peng et al. [17] experimentally analyzed the penetration–explosion effects of conical-shaped charges, and performed numerical simulation using a commercial software (AUTODYN).

Lagrangian and arbitrary Lagrangian and Eulerian (ALE) methods, except the SPH method, involve considerable complexity due to the need for mesh management to handle large deformations of the boundary. Therefore, periodic re-meshing is required to maintain good mesh quality, which increases the computational cost. Although the SPH method has the advantage of handling the large deformation efficiently, it is difficult to accurately represent the boundary surface with this method compared to the Eulerian method. As the metal jet of a shaped charge undergoes large deformation within a very short time and behaves similarly to a compressible fluid flow, it is advantageous to simulate it in an Eulerian framework using modern CFD gas dynamics techniques. Therefore, this study developed a hydrocode to simulate conical- and linear-shaped charges in an Eulerian framework on Cartesian meshes. Elasto-plastic solids were modeled using the CENO scheme [18] and the ghost fluid method [11,12,19] to simulate the large deformation and penetration of a metal liner and a casing. Further, the detonation of a high explosive was simulated using the programmed burn model [10]. For the multi-material interface treatment, the precise boundary conditions between an explosive gas, solids, and void were presented in detail. The developed hydrocode was validated using previously reported numerical and experimental data for 1-D solid impact and the Taylor bar problems.

The main difference between the developed hydrocode and the previous ones is that the present hydrocode newly combines the high-resolution elasto-plastic solid model [11,12] with the detonation model of explosive gas [10] on an Eulerian framework to simulate shaped charge problems. In addition, unlike Chen and Liu [10], since the present hydrocode can be applied regardless of the number of materials, it can solve more practical shaped charge problems.

## 2. Governing Equations

The governing equations for elasto-plastic solids under high-speed and high-strain rates in two-dimensional or axisymmetric geometry in an Eulerian framework can be represented as follows [11,12]:

(1) $$\begin{array}{c} \frac{\partial U}{\partial t}+\frac{\partial F}{\partial x}+\frac{\partial G}{\partial y}=S \end{array}$$

where

(2) $$U=\left(\begin{array}{c} \rho \\ \rho u\\ \rho v\\ \rho E\\ s_{xx}\\ s_{xy}\\ s_{yy} \end{array}\right),\;F=\left(\begin{array}{c} \rho u\\ \rho u^{2}+p\\ \rho uv\\ u\left(\rho E+p\right)\\ us_{xx}\\ us_{xy}\\ us_{yy} \end{array}\right),G=\left(\begin{array}{c} \rho v\\ \rho uv\\ \rho v^{2}+p\\ v\left(\rho E+p\right)\\ vs_{xx}\\ vs_{xy}\\ vs_{yy} \end{array}\right),S=\left(\begin{array}{c} S_{\rho }\\ S_{u}\\ S_{v}\\ S_{E}\\ S_{s_{xx}}\;\\ S_{s_{xy}}\\ S_{s_{yy}} \end{array}\right)$$

where ρ is the density; u and v are the *x*- and *y*-velocity, respectively; E is the specific total energy; p is the pressure; $s_{ij}$ is the deviatoric stress tensor; and S is the source vector. To calculate the temperature change caused by plastic deformation, auxiliary equations for effective plastic strain and temperature are required. The stress tensor, $\sigma_{ij}$, can be divided into a dilatational term, $\left(-p\delta_{ij}\right)$, and a deviatoric term, $s_{ij}$, as

(3) $$\begin{array}{c} \sigma_{ij}=s_{ij}-p\delta_{ij} \end{array}$$

where p is obtained using the Mie–Grüneisen equation-of-state (EOS) and $s_{ij}$ is calculated using the plastic flow theory. This study utilized the Johnson–Cook model [20] as the material model for the elasto-plastic solids. The plastic deformation was calculated using the von Mises J2 flow theory. To consider the plastic deformation of elasto-plastic solids, a radial return mapping algorithm [21,22] was applied. Further details on the governing equations, the auxiliary equations, the material model, and the radial return mapping algorithm appear in Appendix A.

A high-speed explosive gas can be described using the hyperbolic conservation laws of gas dynamics. The governing equation for an explosive gas in two-dimensional or axisymmetric coordinates is

(4) $$\begin{array}{c} \frac{\partial U}{\partial t}+\frac{\partial F}{\partial x}+\frac{\partial G}{\partial y}=S \end{array}$$

where

(5) $$U=\left(\begin{array}{c} \rho \\ \rho u\\ \rho v\\ \rho E \end{array}\right),\;F=\left(\begin{array}{c} \rho u\\ \rho u^{2}+p\\ \rho uv\\ u\left(\rho E+p\right) \end{array}\right),\;G=\left(\begin{array}{c} \rho v\\ \rho uv\\ \rho v^{2}+p\\ v\left(\rho E+p\right) \end{array}\right),\;S=\left(\begin{array}{c} -\frac{\Psi }{x}\rho u\\ -\frac{\Psi }{x}\rho u^{2}\\ -\frac{\Psi }{x}\rho uv\\ -\frac{\Psi }{x}u\left(\rho E+p\right) \end{array}\right)$$

To simulate the detonation process of an explosive charge, a pressure-based programmed burn model [10] was used in the hydrocode developed in this study. The internal energy of the explosive product (gas) was modeled using the Jones–Wilkins–Lee (JWL) EOS. Table 1 shows the parameters of the JWL EOS of TNT and Composition B explosives. More details on the programmed burn model are given in Appendix A.

## 3. Numerical Method

The governing equation for elasto-plastic solids was discretized using the third-order Runge–Kutta method in time and the third-order CENO scheme [18] using the finite difference method and the Lax–Friedrichs flux in space, which is similar to that reported in a previous study [11]. As the CENO scheme uses a component-wise method that does not consider the complicated characteristic decomposition of the governing equations via eigenvalues and eigenvectors, the scheme is somewhat less accurate than the well-known weighted ENO (WENO) scheme [23]. However, this method is easy to implement and requires lower computational costs. The third-order Runge–Kutta scheme and the fifth-order WENO scheme (WENO5) [23] are used to solve the auxiliary equations and the explosive gas equations. The level-set method [24] is used to identify the boundary of each material in a multi-material environment. The details on the numerical methods are reproduced in Appendix B for completeness.

### Ghost Fluid Method

The ghost fluid method is used for boundary conditions at the material interface [11,12,19]. First, the physical properties on the interface are extrapolated around the interface using the following equation:

(6) $$\begin{array}{c} \frac{\partial q}{\partial \tau }+{\vec{V}}_{ext}\cdot \nabla q=0 \end{array}$$

where

(7) $$\begin{array}{c} {\vec{V}}_{ext}=s\frac{\nabla \phi }{\left|\nabla \phi \right|} \end{array}$$

(8) $$\begin{array}{c} s=sign\left(\phi \right) \end{array}$$

Applying the forward Euler method in time and the first-order upwind method in space to Equation (6), the following equation is derived

(9) $$\begin{array}{cc} q_{i,j}^{n+1}=q_{i,j}^{n} & -∆\tau [{\left(sn_{x}\right)}^{+}\left(\frac{q_{i,j}-q_{i-1,j}}{∆x}\right)+{\left(sn_{x}\right)}^{-}\left(\frac{q_{i+1,j}-q_{i,j}}{∆x}\right)\\ & +{\left(sn_{y}\right)}^{+}\left(\frac{q_{i,j}-q_{i,j-1}}{∆y}\right)+{\left(sn_{y}\right)}^{-}\left(\frac{q_{i,j+1}-q_{i,j}}{∆y}\right)] \end{array}$$

where the time step, ∆τ=0.5∆x, is used. Since the purpose of Equation (9) is to populate a thin layer of ghost cells around the interface needed for the numerical method by solving it for a few time steps, it is not necessary to obtain an accurate solution in time and space; the first-order accuracy is sufficient.

**Figure 1:**
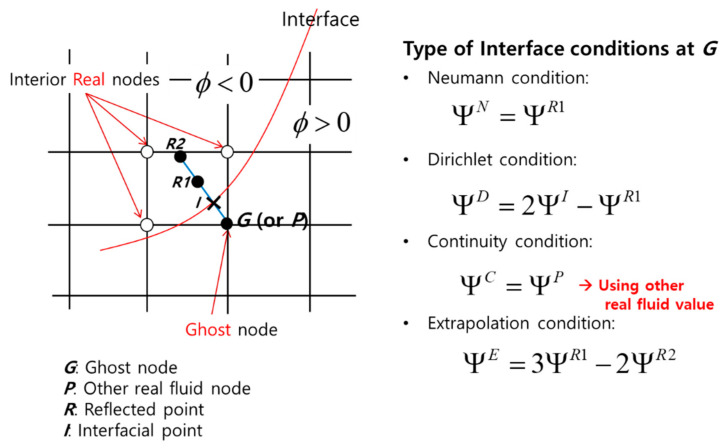
Boundary conditions of the ghost fluid method.

According to the physical variables, there are four boundary conditions: Neumann, Dirichlet, continuity, and extrapolation conditions. The value of the “ghost” node can be calculated from the values of the internal “real” nodes, as shown in Figure 1.

The velocity vector and the stress tensor should be rotated using the local coordinates system on the interface. The unit normal vector, n, the unit tangent vector, t, and the rotation tensor, R, on the local coordinate system are defined as

(10) $$\begin{array}{c} n=\left(n_{x},n_{y}\right) \end{array}$$

(11) $$\begin{array}{c} t=\left(t_{x},t_{y}\right)=\left(n_{y},-n_{x}\right) \end{array}$$

(12) $$\begin{array}{c} R=\left(\begin{array}{cc} n_{x} & n_{y}\\ t_{x} & t_{y} \end{array}\right)=R^{T}=R^{-1} \end{array}$$

Thus, the rotated component in the local coordinate can be calculated using

(13) $$\begin{array}{c} V_{n}=V\cdot n \end{array}$$

(14) $$\begin{array}{c} V_{t}=V\cdot t \end{array}$$

(15) $$\begin{array}{c} {\tilde{\sigma }}_{ij}=R\sigma_{ij}R^{T} \end{array}$$

The values of the “ghost” nodes are imposed according to the type of boundary. In this study, the following four cases were considered.

Solid–solid interface:

(16) $$\begin{array}{c} \rho^{G}=\rho^{N} \end{array}$$

(17) $$\begin{array}{c} V_{n}^{G}=V_{n}^{P} \end{array}$$

(18) $$\begin{array}{c} V_{t}^{G}=V_{t}^{N} \end{array}$$

(19) $$\begin{array}{c} p^{G}=p^{C} \end{array}$$

(20) $$\begin{array}{c} {\tilde{\sigma }}_{nn}^{G}={\tilde{\sigma }}_{nn}^{P} \end{array}$$

(21) $$\begin{array}{c} {\tilde{\sigma }}_{nt}^{G}={\tilde{\sigma }}_{nt}^{P} \end{array}$$

(22) $$\begin{array}{c} {\tilde{\sigma }}_{tt}^{G}={\tilde{\sigma }}_{tt}^{N} \end{array}$$

(23) $$\begin{array}{c} {\bar{\varepsilon }}^{p}{}^{G}={\bar{\varepsilon }}^{p}{}^{E} \end{array}$$

(24) $$\begin{array}{c} T^{G}=T^{C} \end{array}$$

Solid–void (or gas–void) interface:

(25) $$\begin{array}{c} \rho^{G}=\rho^{N} \end{array}$$

(26) $$\begin{array}{c} V_{n}^{G}=V_{n}^{N} \end{array}$$

(27) $$\begin{array}{c} V_{t}^{G}=V_{t}^{N} \end{array}$$

(28) $$\begin{array}{c} p^{G}=p^{D} \end{array}$$

(29) $$\begin{array}{c} {\tilde{\sigma }}_{nn}^{G}={\tilde{\sigma }}_{nn}^{D} \end{array}$$

(30) $$\begin{array}{c} {\tilde{\sigma }}_{nt}^{G}={\tilde{\sigma }}_{nt}^{D} \end{array}$$

(31) $$\begin{array}{c} {\tilde{\sigma }}_{tt}^{G}={\tilde{\sigma }}_{tt}^{N} \end{array}$$

(32) $$\begin{array}{c} {\bar{\varepsilon }}^{p}{}^{G}={\bar{\varepsilon }}^{p}{}^{E} \end{array}$$

(33) $$\begin{array}{c} T^{G}=T^{E} \end{array}$$

Solid–gas interface:

(34) $$\begin{array}{c} \rho^{G}=\rho^{N} \end{array}$$

(35) $$\begin{array}{c} V_{n}^{G}=V_{n}^{C} \end{array}$$

(36) $$\begin{array}{c} V_{t}^{G}=V_{t}^{N} \end{array}$$

(37) $$\begin{array}{c} p^{G}=p^{P} \end{array}$$

(38) $$\begin{array}{c} {\tilde{\sigma }}_{nn}^{G}=-p^{P} \end{array}$$

(39) $$\begin{array}{c} {\tilde{\sigma }}_{nt}^{G}=0 \end{array}$$

(40) $$\begin{array}{c} {\tilde{\sigma }}_{tt}^{G}={\tilde{\sigma }}_{tt}^{N} \end{array}$$

(41) $$\begin{array}{c} {\bar{\varepsilon }}^{p}{}^{G}={\bar{\varepsilon }}^{p}{}^{E} \end{array}$$

(42) $$\begin{array}{c} T^{G}=T^{E} \end{array}$$

Gas–solid interface:

(43) $$\begin{array}{c} \rho^{G}=\rho^{N} \end{array}$$

(44) $$\begin{array}{c} V_{t}^{G}=V_{t}^{N} \end{array}$$

(45) $$\begin{array}{c} e^{G}=e^{N} \end{array}$$

(46) $$\begin{array}{c} V_{n}^{G}=\{\begin{array}{c} V_{n}^{P},\;\mathrm{if}\;V_{n}^{R1}>V_{n}^{P}>0\;\mathrm{or}\;V_{n}^{P}<V_{n}^{R1}\leq 0\;\mathrm{or}\;{\left(V_{n}^{R1}>0\;\mathrm{and}\;V_{n}^{P}\leq 0\right)}^{P}\;\\ V_{n}^{R},\;\mathrm{otherwise} \end{array} \end{array}$$

(47) $$\begin{array}{c} p^{G}=\{\begin{array}{c} p\left(\rho^{G},e^{G}\right),\;\mathrm{if}\;p^{P}>0\;\\ 0,\;\mathrm{otherwise} \end{array} \end{array}$$

The “real” nodes values, R1 and R2, were calculated by interpolating from the values of the surrounding internal nodes. The interpolation function can be expressed as

(48) $$\begin{array}{c} \Psi =a_{1}+a_{2}x+a_{3}y+a_{4}xy \end{array}$$

where a is the coefficients. If there are four neighboring nodes around R1 and R2, including the boundary, the bilinear interpolation is used. For the Dirichlet condition, the coefficients are calculated as

(49) $$\left(\begin{array}{cccc} 1 & x_{1} & y_{1} & x_{1}y_{1}\\ 1 & x_{2} & y_{2} & x_{2}y_{2}\\ 1 & x_{3} & y_{3} & x_{3}y_{3}\\ 1 & x_{I} & y_{I} & x_{I}y_{I} \end{array}\right)\left(\begin{array}{c} a_{1}\\ a_{2}\\ a_{3}\\ a_{4} \end{array}\right)=\left(\begin{array}{c} \Psi_{1}\\ \Psi_{2}\\ \Psi_{3}\\ \Psi^{I} \end{array}\right)$$

For the Neumann condition, the coefficients can be expressed as

(50) $$\left(\begin{array}{cccc} 1 & x_{1} & y_{1} & x_{1}y_{1}\\ 1 & x_{2} & y_{2} & x_{2}y_{2}\\ 1 & x_{3} & y_{3} & x_{3}y_{3}\\ 0 & n_{x} & n_{y} & n_{x}y_{I}+n_{y}x_{I} \end{array}\right)\left(\begin{array}{c} a_{1}\\ a_{2}\\ a_{3}\\ a_{4} \end{array}\right)=\left(\begin{array}{c} \Psi_{1}\\ \Psi_{2}\\ \Psi_{3}\\ \partial \Psi^{I}/\partial n \end{array}\right)$$

If there are less than four neighboring nodes around R1 or R2, the least squared interpolation is used, which can be expressed as

(51) $$\left(\begin{array}{cccc} \sum 1 & \sum x & \sum y & \sum xy\\ \sum x & \sum x^{2} & \sum xy & \sum x^{2}y\\ \sum y & \sum xy & \sum y^{2} & \sum xy^{2}\\ \sum xy & \sum x^{2}y & \sum xy^{2} & \sum x^{2}y^{2} \end{array}\right)\left(\begin{array}{c} a_{1}\\ a_{2}\\ a_{3}\\ a_{4} \end{array}\right)=\left(\begin{array}{c} \sum \Psi \\ \sum x\Psi \\ \sum y\Psi \\ \sum xy\Psi \end{array}\right)$$

To determine whether two materials collide or not, the following condition is used [12]:

(52) $$\begin{array}{c} \left|\phi_{1}+\phi_{2}\right|<\varepsilon \end{array}$$

where $\phi_{1}$ and $\phi_{2}$ are the level-set functions of two materials and ε=CFL⋅∆x.

As the deviatoric stress tensor in the “ghost” node obtained using the ghost fluid method does not satisfy the $J_{1}$ invariant condition, $J_{1}\equiv s_{nn}+s_{tt}=0$, the stress tensor can be corrected as

(53) $$\begin{array}{c} {\tilde{\sigma }}_{tt}^{G}=-{\tilde{\sigma }}_{nn}^{G}-2p^{G} \end{array}$$

The overall procedure of the present method is summarized in Algorithm 1.
**Algorithm 1** The numerical procedure for the present hydrocode.1:Generate mesh and setup initial conditions.2:Moving level-set fields, $\phi_{i}$, using the level-set method.3:Populate the ghost cells around the interface by solving Equation (9).4:Impose boundary conditions according to the type of boundary by using the ghost fluid method.5:Solve the governing equation (Equation (1)) to obtain the elastic predictor step for solids.6:Apply the radial return mapping algorithm to perform the plastic corrector step.7:Solve the auxiliary equations for the temperature of the solids.8:Apply the programmed burn model to the explosive charge.9:Solve the governing equation (Equation (4)) for explosive gas.10:Update the time step.11:Go back to step 2 and repeat until the last time step is reached.

## 4. Results and Discussion

### 4.1. One-Dimensional Shockwave Propagation in Elasto-Plastic Solid

**Figure 2:**
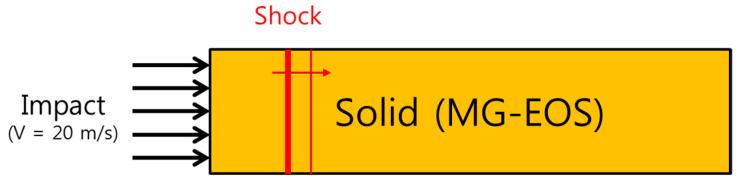
Shockwave propagation in solids by impact.

This problem considered the propagation of shockwaves in a solid after a 20 m/s impact on one side in one dimension (Figure 2). Table 2 shows the parameters of the Mie–Grüneisen EOS of the solid used in this problem.

**Figure 3:**
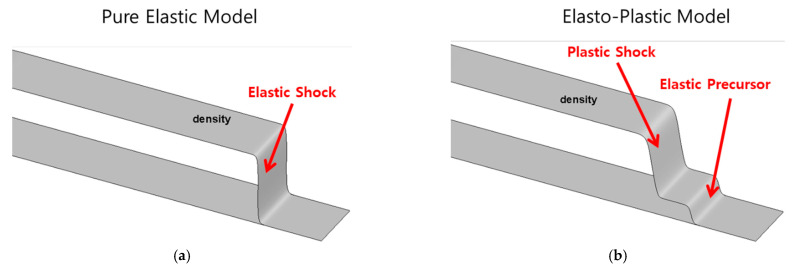
Computed shockwave propagation in an (**a**) elastic and (**b**) elasto-plastic solid by a 20 m/s impact at the left end.

Figure 3 shows the computed shockwave propagations of the elastic and elasto-plastic solids. As shown in Figure 3a, only one shockwave propagates inside the solid in a pure elastic solid; however, in an elasto-plastic solid, first, a weak elastic wave known as an elastic precursor propagates, after which a strong plastic wave propagates, as shown in Figure 3b. The comparison of these results to those by Udaykumar et al. [25] in Table 3 indicates that the two results are consistent.

### 4.2. Taylor Bar Impact Problem

**Figure 4:**
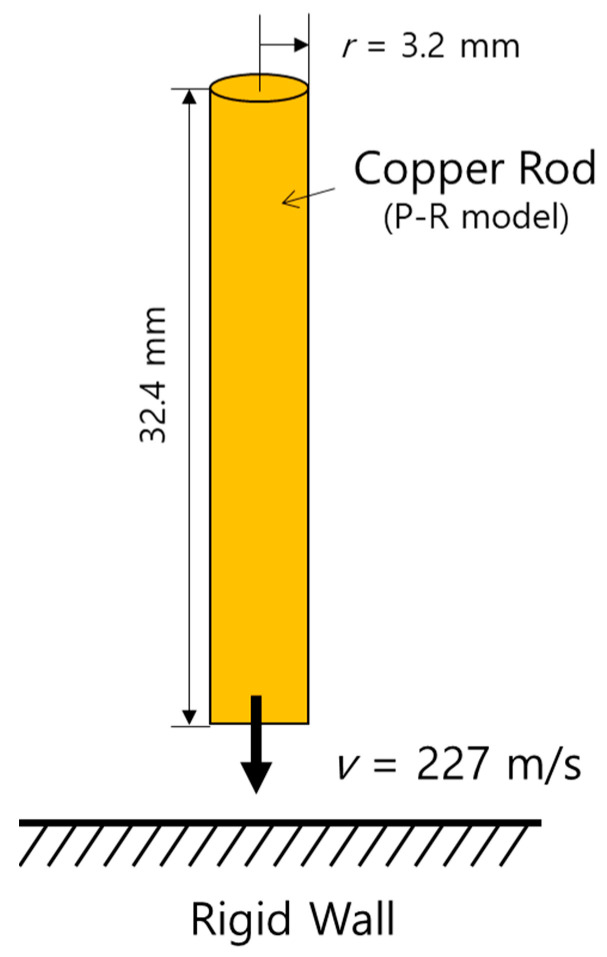
Schematic of the Taylor bar impact problem.

The Taylor bar problem, which has been simulated by numerous researchers, considers that a conical copper rod collides with a rigid wall at high speed (227 m/s), as shown in Figure 4. The copper rod has an initial radius of 3.2 mm and a length of 32.4 mm, and is considered an elasto-plastic material.

**Figure 5:**
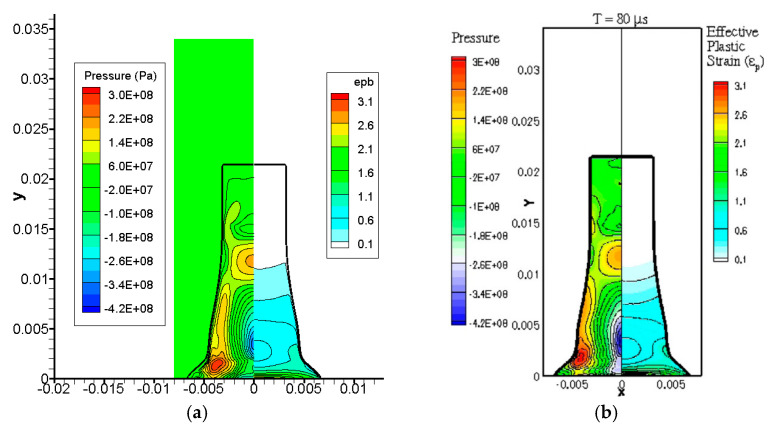
Interface shape and the contours of the pressure and effective plastic strain in the copper rod after collision at t=80 μs. Results of (**a**) this study and (**b**) Sambasivan et al. [12]. Reprinted with permission from Elsevier.

Figure 5 shows the comparison of the numerical results of the pressure and effective plastic strain in the copper rod obtained in this study at t=80 μs to those obtained by Sambasivan et al. [12], where the deformed shape of the rod and the inner contour plots are similar to each other. Table 4 summarizes the comparison of the computed results of the final geometry of the copper rod obtained in previous studies to those obtained in this study. Although the final shape may exhibit slightly different computational results depending on the physical model and numerical technique used, the computed results of this study were consistent with those of previous studies within a reasonable error range. The computed results for the maximum ${\bar{\varepsilon }}_{p}$ show slight differences from those of previous researchers. As an analysis of the causes of these differences is beyond the scope of this study, further comparison and analysis of the different models and numerical methods may improve the present method in future studies.

### 4.3. Generation and Penetration of a High-Speed Metal Jet

**Figure 6:**
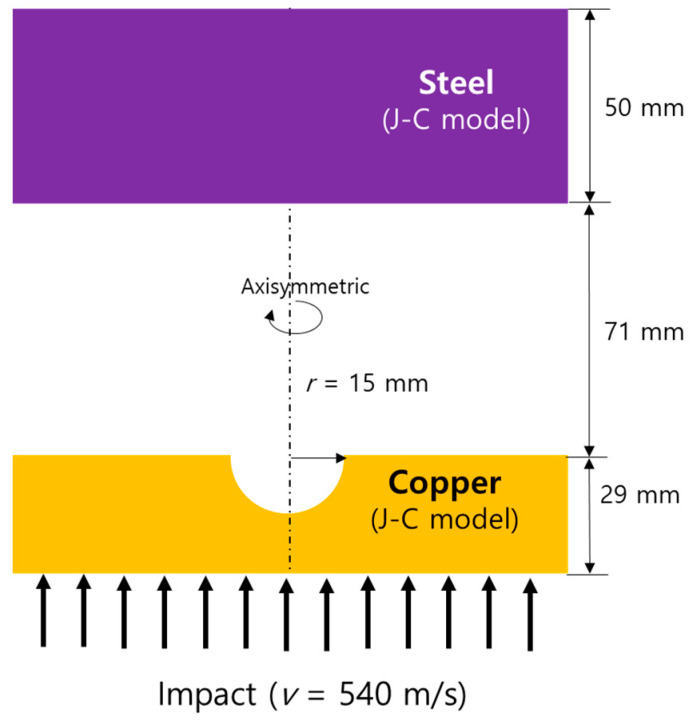
Schematic of the high-speed copper jet penetrating steel problem.

This problem was designed to verify whether the present hydrocode can successfully simulate the generation of a metal jet and the penetration of the target material. Figure 6 shows a schematic of the problem, where a strong impact (v=540 m/s) was applied to the bottom of the hemispherical groove of the copper liner with a thickness of 29 mm and a radius of 15 mm, and a 50 mm thick steel plate was placed 71 mm apart from the copper plate. The high-speed metal jet was created by a shockwave after a strong impact to the opposite side of the copper liner with a hemispherical groove, which is a similar mechanism to that of the jet generation of shaped charges.

**Figure 7:**
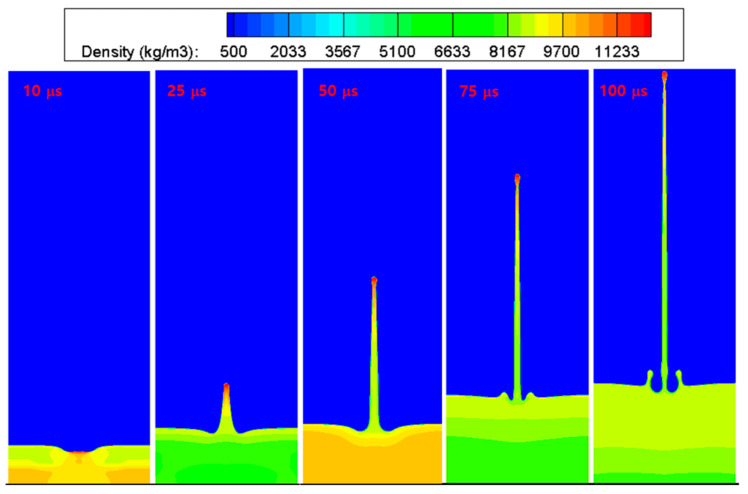
Evolution of the metal jet by the impact of a hemispherical groove in copper. The interface shapes and density contours at five time instants up to 100 μs after impact are shown.

**Figure 8:**
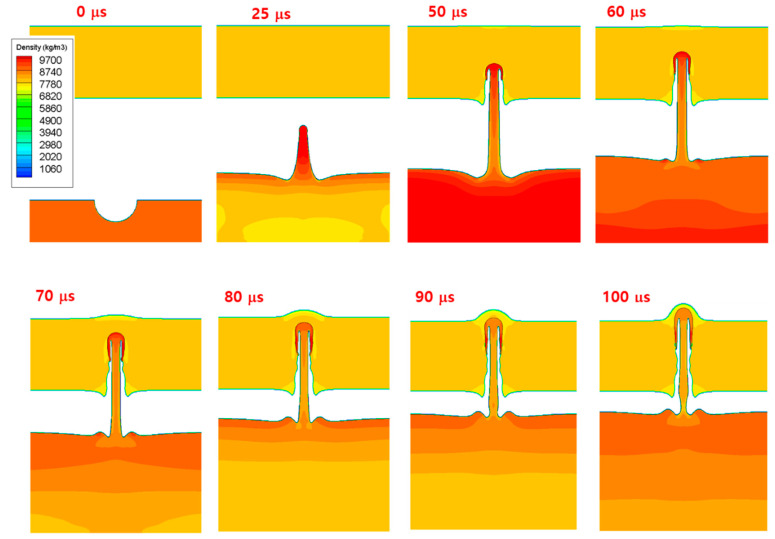
Computed results of the penetration of steel by the copper jet generated from the hemispherical groove. The interface shapes and density contours at eight time instants up to 100 μs after impact are shown.

Figure 7 shows the computed interface shapes and density contours at five time instants up to 100 μs after impact, where the generation and growth of the copper jet were well simulated. The results of this study on the evolution of the interface were similar to those of previous studies [12,29]. Figure 8 shows the interface shapes and density contours plotted at eight time instants up to 100 μs after impact. The image revealed that the current hydrocode can successfully simulate the solid penetration process by a high-speed metal jet.

### 4.4. Conical-Shaped Charge

**Figure 9:**
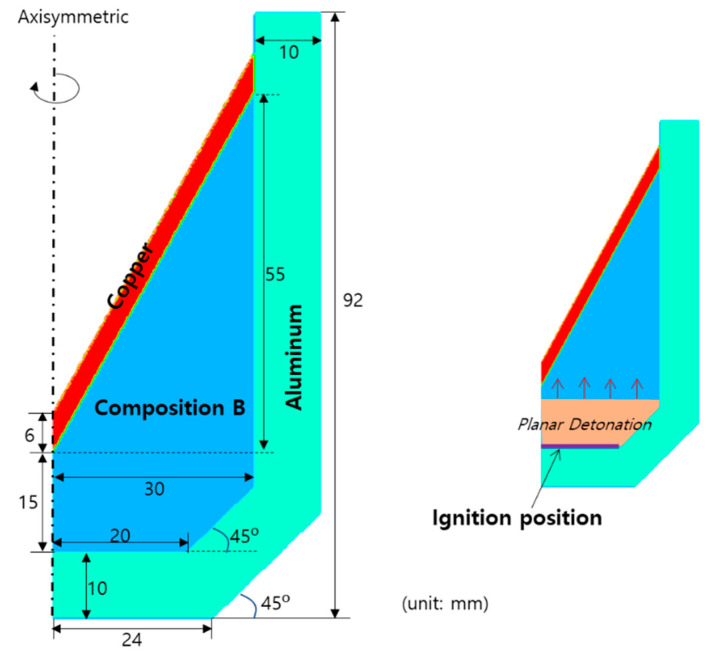
Schematic of a conical shaped charge problem.

A conical-shaped charge problem was numerically simulated using the developed hydrocode to examine the detonation of a high explosive and the resulting jet of metal liner. Figure 9 shows the schematic of this problem. Composition B, copper, and aluminum were used for the explosive charge, liner, and casing, respectively. The explosive was assumed to detonate from the bottom plane.

**Figure 10:**
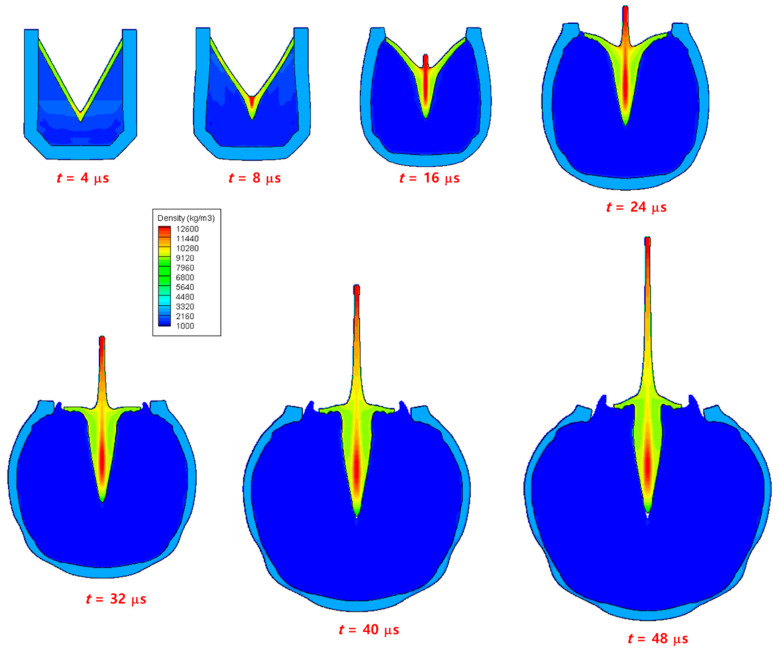
Snapshots of the jet evolution of a conical-shaped charge. The interface shapes and density contours are plotted at seven time instants up to 48 μs after detonation.

**Figure 11:**
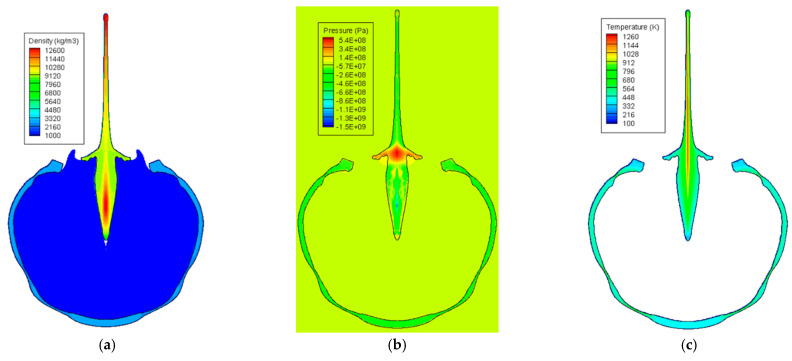
Metal jet of a conical-shaped charge at t=52 μs: (**a**) density contour, (**b**) pressure contour, and (**c**) temperature contour.

Figure 10 shows the expansion of the explosive gas and the evolution of a metal jet by the detonation of an explosive charge at seven different time instants. As the detonation wave propagated, the pressure was concentrated in the center of the copper liner, and a jet began to form. As the wave propagation proceeded, a high-speed thin jet was formed in the front of the liner, the lateral wing of the liner contracted towards the center, and a very slow slug that occupied most of the mass emerged in the rear part of the liner. In addition, the angle of the lateral liner wing gradually increased, and became horizontal (90°) at approximately t=32 μs, after which it is formed in the reverse direction. In addition, the aluminum case expanded owing to the pressure of the detonation wave, and the thickness of the case reduced at a specific part. Figure 11 shows the density, pressure, and temperature contours at t=52 μs after detonation, where the density of the liner was high at the front of the jet and the center of the slug, most of the pressure was concentrated in the central part of the liner, and the temperature was high along the central axis of the liner. At t=52 μs, the maximum density, pressure, and temperature of the jet were $15,342\;\mathrm{kg}/m^{3}$, 610 MPa, and 1260 K, respectively. The evolution of the jet demonstrated in this study was consistent to those of previous experiments and simulations [4,5,6,7,9,10,30,31,32], indicating that the hydrocode developed in this study can successfully simulate the conical-shaped charge.

### 4.5. Linear-Shaped Charge

**Figure 12:**
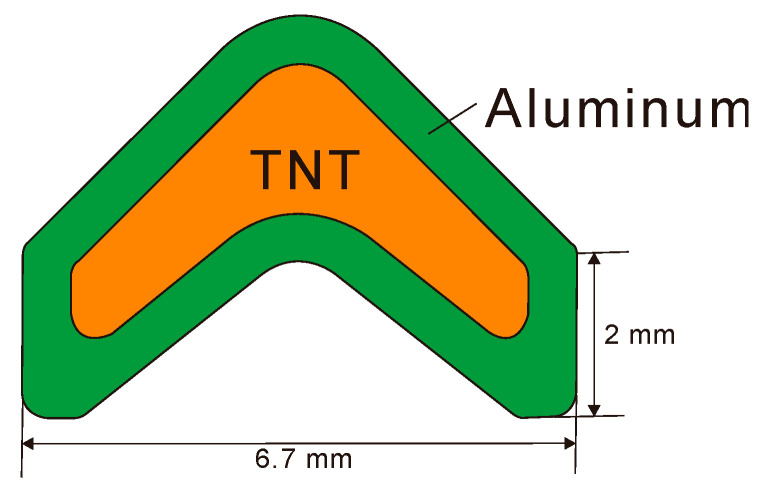
Schematic of a linear-shaped charge problem.

Figure 12 shows a linear-shaped charge problem, where an aluminum liner, which was also used as the casing of the explosive charge with a thickness of 0.58 mm, surrounds a TNT explosive. As the linear-shaped charge has a two-dimensional geometry, compared to the conical-shaped charge, the cross-section of the explosive of the linear-shaped charge detonated at the same time. In addition, as there was no additional casing metal, which is usually thicker than the liner, in the linear-shaped charge, the case was destroyed earlier than in the conical-shaped charge, thus resulting in the rapid release of the pressure of the explosive gas to the external environment. Furthermore, the length of the jet of the linear-shaped charge was significantly shorter than that of the conical charge owing to the two-dimensional geometry and the pressure loss of the linear-shaped charge. Because of these characteristics, the linear-shaped charge is generally more difficult to numerically simulate than the conical-shaped charge.

**Figure 13:**
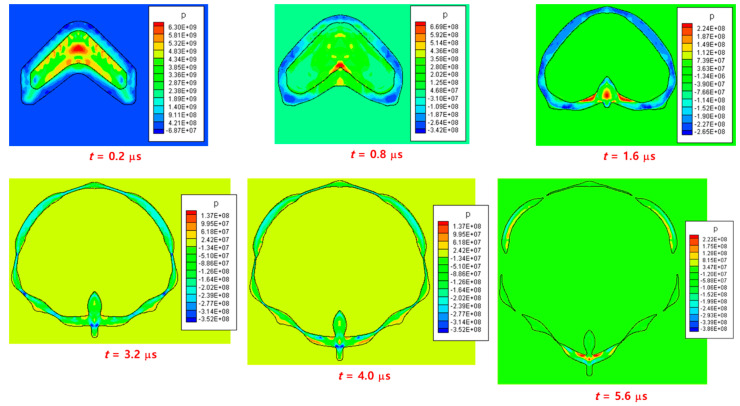
Snapshots of the jet evolution of a linear-shaped charge. The interface shapes and pressure contours are plotted at six time instants up to 5.6 μs after detonation.

**Figure 14:**
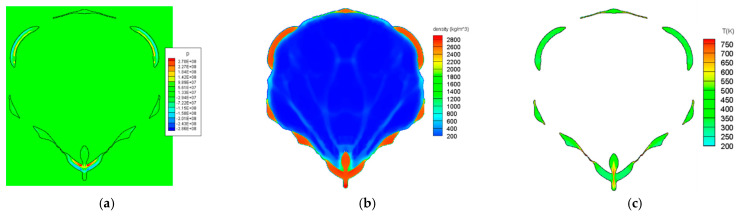
Jet formation of a linear-shaped charge at t=7 μs: (**a**) pressure contour, (**b**) density contour, and (**c**) temperature contour.

Figure 13 shows the expansion of the explosive gas by the detonation, the deformation of the liner, and the formation of the jet. Most of the pressure generated by the two-dimensional detonation was concentrated in the center of the liner to create a jet, and some was used to expand the other part of the liner. Over time, a high-speed jet was formed in the front of the liner and a low-speed slug in the rear part of the liner. The pressure of the detonation wave reduced the thickness of a specific part of the case, and some parts were fragmented at approximately t=5.6 μs. In addition, the length of the jet was considerably shorter than that of the conical-shaped charge, and the surrounding casing moved forward together as the jet developed, and eventually, only the jet part was separated from the casing. Figure 14 shows the pressure, density, and temperature contours at t=7 μs after detonation. The casing was fragmented in several parts, through which the explosive gas escaped. In addition, most of the pressure was concentrated on the central wing portion of the jet, indicating a high temperature along the central axis of the jet. At this time, the jet was almost separated from the surrounding material and moved independently. These results are consistent with the experimental and numerical results of previous studies [13,33,34], indicating that the developed hydrocode can successfully simulate the linear-shaped charge.

## 5. Conclusions

In this study, a conical- and a linear-shaped charge were numerically simulated using an Eulerian multi-material and multi-phase flow model for elasto-plastic solids and a high-explosive gas with a detonation. The elasto-plastic solids and the detonation of a high explosive charge were modeled using the Johnson–Cook material model and the programmed burn model, respectively. In addition, the stress was corrected back to the yield surface using the radial return mapping algorithm. The model was solved on the Cartesian grids using a third-order CENO and the third-order Runge–Kutta method for the elasto-plastic solids, and using a fifth-order WENO and the third-order Runge–Kutta method for the constitutive equations and the explosive gas equations. The boundary conditions on the material interfaces were imposed depending on the two materials crossing the boundary using the level-set and ghost fluid methods. The developed hydrocode was validated using two high-speed impact problems, and the hydrocode was used to successfully simulate the evolution and penetration of metal jets in the shaped charges after the detonation of an explosive charge. The developed hydrocode is expected to help in the simulation of large deformation and penetration problems of elasto-plastic solids other than shaped charges. In addition, future studies will investigate shaped charges with a three-dimensional geometry, which may require adaptive mesh refinements and parallel processing techniques.

## Figures and Tables

**Table 1:** Jones–Wilkins–Lee (JWL) equation-of-state (EOS) parameters of TNT and Composition B.

	$\rho_{0}\;\left(\mathrm{kg}/m^{3}\right)$	$p_{CJ}\;\left(\mathrm{GPa}\right)$	$v_{d}\;\left(m/s\right)$	A (GPa)	B (GPa)	$R_{1}$	$R_{2}$	ω	$I_{0}\;\left(J/\mathrm{kg}\right)$
TNT	1630	21	6930	371.2	3.210	4.15	0.95	0.30	$4.29\times 10^{6}$
Composition B	1717	29	7980	524.2	7.678	4.20	1.10	0.34	$4.95\times 10^{6}$

**Table 2:** Parameters of the Mie–Grüneisen EOS.

Γ	s	$c_{0}\;(m/s)$	$\rho_{0}\;(\mathrm{kg}/m^{3})$	$e_{0}\;(J/\mathrm{kg})$	G (GPa)	Y (MPa)
2.0	1.49	3940	8930	110,920	45	90

**Table 3:** Comparison of the results of this study to those of Udaykumar et al. [25].

	Elastic Precursor		Plastic Shock	
	Udaykumar et al. [25]	Present	Udaykumar et al. [25]	Present
Density ($\mathrm{kg}/m^{3}$)	8938.9	8939.8	8973.5	8973.3

**Table 4:** Comparison of the results of the final geometry of the copper rod in this study to those of previous studies.

	Final Height (mm)	Final Base Radius (mm)	$\mathrm{Maximum}\;{\bar{\varepsilon }}_{p}$
Present	21.44	6.65	2.672
Sambasivan et al. [12]	21.53	7.05	3.169
Mehmandoust and Pishevar [26]	21.80	6.53	2.178
Tran and Udaykumar [27]	21.15	7.15	2.86
Udaykumar et al. [25]	21.4	6.97–7.24	-
Camacho and Ortiz [28]	21.42–21.44	7.21–7.24	2.97–3.25

## Data Availability

Not applicable.

## Appendix A. Governing Equations

### Appendix A.1. Eulerian Elasto-Plastic Solid Model

The governing equations for elasto-plastic solids under high-speed and high-strain rates in two-dimensional or axisymmetric geometry in an Eulerian framework can be represented as follows [11,12]:

(A1) $$\begin{array}{c} \frac{\partial U}{\partial t}+\frac{\partial F}{\partial x}+\frac{\partial G}{\partial y}=S \end{array}$$

where

(A2) $$U=\left(\begin{array}{c} \rho \\ \rho u\\ \rho v\\ \rho E\\ s_{xx}\\ s_{xy}\\ s_{yy} \end{array}\right),F=\left(\begin{array}{c} \rho u\\ \rho u^{2}+p\\ \rho uv\\ u\left(\rho E+p\right)\\ us_{xx}\\ us_{xy}\\ us_{yy} \end{array}\right),G=\left(\begin{array}{c} \rho v\\ \rho uv\\ \rho v^{2}+p\\ v\left(\rho E+p\right)\\ vs_{xx}\\ vs_{xy}\\ vs_{yy} \end{array}\right),S=\left(\begin{array}{c} S_{\rho }\\ S_{u}\\ S_{v}\\ S_{E}\\ S_{s_{xx}}\;\\ S_{s_{xy}}\\ S_{s_{yy}} \end{array}\right)$$

where ρ is the density; u and v are the *x*- and *y*-velocity, respectively; E is the specific total energy; p is the pressure; and $s_{ij}$ is the deviatoric stress tensor. The source terms in the vector S are given as follows:

(A3) $$\begin{array}{c} S_{\rho }=-\frac{\Psi }{x}\rho u \end{array}$$

(A4) $$\begin{array}{c} S_{u}=\frac{\partial s_{xx}}{\partial x}+\frac{\partial s_{xy}}{\partial y}+\frac{\Psi \;}{x}\left(2s_{xx}+s_{yy}-\rho u^{2}\right) \end{array}$$

(A5) $$\begin{array}{c} S_{v}=\frac{\partial s_{xy}}{\partial x}+\frac{\partial s_{yy}}{\partial y}+\frac{\Psi \;}{x}\left(s_{xy}-\rho uv\right) \end{array}$$

(A6) $$\begin{array}{c} S_{E}=\frac{\partial }{\partial x}\left(us_{xx}+vs_{xy}\right)+\frac{\partial }{\partial y}\left(us_{xy}+vs_{yy}\right)+\frac{\Psi }{x}\left[us_{xx}+vs_{xy}-u\left(\rho E+p\right)\right] \end{array}$$

(A7) $$\begin{array}{c} S_{s_{xx}}=s_{xx}\left(\frac{\partial u}{\partial x}+\frac{\partial v}{\partial y}\right)+2\Omega_{xy}s_{xy}+2G\left(\frac{\partial u}{\partial x}-\Phi \right) \end{array}$$

(A8) $$\begin{array}{c} S_{s_{xy}}=s_{xy}\left(\frac{\partial u}{\partial x}+\frac{\partial v}{\partial y}\right)+\Omega_{xy}\left(s_{yy}-s_{xx}\right)+G\left(\frac{\partial u}{\partial y}+\frac{\partial v}{\partial x}\right) \end{array}$$

(A9) $$\begin{array}{c} S_{s_{yy}}=s_{yy}\left(\frac{\partial u}{\partial x}+\frac{\partial v}{\partial y}\right)-2\Omega_{xy}s_{xy}+2G\left(\frac{\partial v}{\partial y}-\Phi \right) \end{array}$$

where G is the shear modulus, $\Omega_{xy}$ is the spin tensor, and Ψ is the switch function, and can be expressed as

(A10) $$\begin{array}{c} \Omega_{xy}=\frac{1}{2}\left(\frac{\partial u}{\partial y}-\frac{\partial v}{\partial x}\right) \end{array}$$

(A11) $$\begin{array}{c} \Psi =\{\begin{array}{c} 0,\;\mathrm{for}\;2D\\ 1,\;\mathrm{for}\;\mathrm{axisymmetric} \end{array} \end{array}$$

(A12) $$\begin{array}{c} \Phi =\frac{1}{3}\left(\frac{\partial u}{\partial x}+\frac{\partial v}{\partial y}+\frac{\Psi u}{x}\right) \end{array}$$

To calculate the temperature change caused by plastic deformation, auxiliary equations for effective plastic strain and temperature are required. As shockwave propagation and the plastic deformation process of a shaped charge occur within a short time, the temperature change in a solid could be mainly attributed to plastic deformation, and the other effects, such as heat transfer, are negligible. The resulting auxiliary equations are given as

(A13) $$\begin{array}{c} \frac{\partial {\bar{\epsilon }}^{p}}{\partial t}+u\frac{\partial {\bar{\epsilon }}^{p}}{\partial x}+v\frac{\partial {\bar{\epsilon }}^{p}}{\partial y}={\dot{\bar{\epsilon }}}^{p} \end{array}$$

(A14) $$\begin{array}{c} \frac{\partial T}{\partial t}+u\frac{\partial T}{\partial x}+v\frac{\partial T}{\partial y}=\frac{\beta {\dot{W}}_{p}}{\rho C_{v}}\; \end{array}$$

The effective plastic strain rate, ${\dot{\bar{\epsilon }}}^{p}$, in the aforementioned equation can be expressed as

(A15) $$\begin{array}{c} {\dot{\bar{\epsilon }}}^{p}=\sqrt{\frac{2}{3}}\Lambda \end{array}$$

where the consistency parameters Λ and ξ can be expressed as

(A16) $$\begin{array}{c} \Lambda =\frac{\xi }{∆t} \end{array}$$

(A17) $$\begin{array}{c} \xi =\frac{\sqrt{s_{ij,tr}s_{ij,tr}}-\sqrt{2/3}\sigma_{Y}^{0}}{2G+\left(2/3\right)h} \end{array}$$

where ∆t is the time step, $s_{ij,tr}$ is the trial deviatoric stress, $\sigma_{Y}^{0}$ is the yield stress at a previous time step, and h is the hardening coefficient. The stress power caused by the plastic work, ${\dot{W}}_{p}$, is computed as

(A18) $$\begin{array}{c} {\dot{W}}_{p}={\dot{\bar{\epsilon }}}^{p}s_{e} \end{array}$$

where $s_{e}$ is the von Mises effective stress. The Taylor–Quinney (T–Q) parameter, β, which indicates the fraction of mechanical power converted to thermal power, is taken as

(A19) $$\begin{array}{c} \beta =0.9 \end{array}$$

Experimental results of Soares and Hokka [35] show that the T–Q parameter of some materials, such as Ti, Fe, Cu, and Sn, have values of approximately 0.8–0.9 under sufficient plastic strain. The stress tensor, $\sigma_{ij}$, can be divided into a dilatational term, $\left(-p\delta_{ij}\right)$, and a deviatoric term, $s_{ij}$, as

(A20) $$\begin{array}{c} \sigma_{ij}=s_{ij}-p\delta_{ij} \end{array}$$

where p is obtained using the Mie–Grüneisen equation-of-state (EOS) and $s_{ij}$ is calculated using the plastic flow theory.

### Appendix A.2. Material Model

The Johnson–Cook model [20] is expressed as

(A21) $$\begin{array}{c} \sigma_{Y}=\left[A+B{\left({\bar{\epsilon }}^{p}\right)}^{n}\right]\left[1+C\;\ln\left(\frac{{\dot{\bar{\epsilon }}}^{p}}{{\dot{\bar{\epsilon }}}_{0}^{p}}\right)\right]\left[1-{\left(\frac{T-T_{0}}{T_{m}-T_{0}}\right)}^{m}\right] \end{array}$$

where $0<{\dot{\bar{\epsilon }}}^{p}\leq {\dot{\bar{\epsilon }}}_{0}^{p}$ and $T_{0}\leq T<T_{m}$. $T_{0}$ and $T_{m}$ are the reference room and melting temperatures of materials, respectively. Compared to the Prandtl–Ruess model [36], which considers only the rate-independent strain hardening effect (the first term), the Johnson–Cook model considers the rate-dependent strain hardening effect (the second term) and thermal softening effect (the third term).

### Appendix A.3. Radial Return Mapping Algorithm

The following yield function $f\left(s_{ij},\sigma_{Y}\right)$ was introduced to determine whether a solid is in a pure elastic (f≤0) or plastic (f>0) domain:

(A22) $$\begin{array}{c} f\left(s_{ij},\sigma_{Y}\right)=s_{e}-\sigma_{Y}=0 \end{array}$$

where

(A23) $$\begin{array}{c} s_{e}=\sqrt{\frac{3}{2}s_{ij}s_{ij}}=\{\begin{array}{c} \sqrt{\frac{3}{2}\left(s_{xx}^{2}+s_{yy}^{2}+2s_{xy}^{2}\right)},\;\mathrm{for}\;2D\\ \sqrt{3\left(s_{xx}^{2}+s_{yy}^{2}+s_{xy}^{2}+s_{xx}s_{yy}\right)},\;\mathrm{for}\;\mathrm{axisymmetric} \end{array} \end{array}$$

To consider the plastic deformation of elasto-plastic solids, a radial return mapping algorithm [21,22] was applied. This algorithm can be divided into two steps. In the first step, the effective stress is calculated by assuming that the solid is a perfectly elastic material. In the second step, if the calculated effective stress is less than the yield stress, the effective stress is used; otherwise, it is corrected back to the yield surface using the stress. If the stress calculated in the first step is denoted as the “trial” stress, $s_{ij,tr}$, and the stress corrected in the second step is denoted as the “corrected” stress, $s_{ij,cor}$, the resulting stress can be obtained using

(A24) $$\begin{array}{c} s_{ij}=\{\begin{array}{cc} s_{ij,tr}, & \;\mathrm{if}\;s_{e,tr}\leq \sigma_{Y}\\ s_{ij,cor}, & \;\mathrm{otherwise} \end{array} \end{array}$$

where

(A25) $$\begin{array}{c} s_{ij,cor}=\left[1-\frac{s_{e,tr}-\sigma_{Y}^{0}}{s_{e,tr}\left(1+\frac{h}{3G}\right)}\right]s_{ij,tr} \end{array}$$

### Appendix A.4. Mie–Grüneisen EOS for Solids

The dilatational stress (pressure) of solids was modeled using the Mie–Grüneisen [37,38] EOS, which can be expressed as

(A26) $$\begin{array}{c} p=\Gamma \rho e+f\left(\rho \right) \end{array}$$

To prevent the square of the sound speed from becoming negative, Equation (A26) can be rewritten as follows:

(A27) $$\begin{array}{c} p=\{\begin{array}{c} p_{0}+\Gamma_{0}\rho_{0}\left(e-e_{0}\right)+\frac{c_{0}^{2}\rho_{0}\left(\rho -\rho_{0}\right)\left[2\rho -\Gamma_{0}\left(\rho -\rho_{0}\right)\right]}{2{\left[\rho -s\left(\rho -\rho_{0}\right)\right]}^{2}},\;\mathrm{if}\;\rho \geq \rho_{0}\\ p_{0}+\Gamma_{0}\rho_{0}\left(e-e_{0}\right)+c_{0}^{2}\rho_{0}\left(\rho -\rho_{0}\right),\;\mathrm{otherwise} \end{array} \end{array}$$

where $p_{0}$ is the reference pressure, $e_{0}$ is the reference internal energy, $\rho_{0}$ is the density of unstressed material, $c_{0}$ is the bulk sound speed at zero pressure, $\Gamma_{0}$ is the Grüneisen parameter, and s is the coefficient that relates the shock speed to the particle velocity.

The sound speed of compressible solids, c, is given as

(A28) $$\begin{array}{c} c^{2}=\{\begin{array}{cc} \frac{\Gamma_{0}\rho_{0}p}{\rho^{2}}+\frac{c_{0}^{2}\rho_{0}\left[\rho +\left(s-\Gamma_{0}\right)\left(\rho -\rho_{0}\right)\right]}{{\left[\rho -s\left(\rho -\rho_{0}\right)\right]}^{3}} & ,\;\mathrm{if}\;\rho \geq \rho_{0}\\ \;\frac{\Gamma_{0}\rho_{0}p}{\rho^{2}}+c_{0}^{2} & ,\;\mathrm{otherwise} \end{array} \end{array}$$

### Appendix A.5. Programmed Burn Model

The programmed burn model based on the theory of detonation shock dynamics assumes that a detonation wave propagates at a constant velocity after the detonation. This constant velocity is known as the detonation velocity, and its value is determined experimentally according to the type of explosive. As the detonation wave propagates, the explosive power is burned and turned into an explosive gas, thus releasing its internal energy. In the programmed burn model, the internal energy of the explosive was calculated by introducing the Heaviside function located at the front of the detonation wave, which can be expressed as

(A29) $$\begin{array}{c} e=e_{0}\left(1-H\right)+e_{product}\left(\frac{p}{F},\rho \right)H \end{array}$$

where H is the Heaviside function and $e_{0}$ is the initial internal energy. The internal energy of the explosive product (gas), $e_{product}$, was modeled using the Jones–Wilkins–Lee (JWL) EOS. The programmed burn model introduces a combustion function, F, which has a value of 0 when no explosive is burned and a value of 1 when completely combusted, and this value varies smoothly for several computational cells around the front of the detonation wave. The combustion function used in this study is

(A30) $$\begin{array}{c} F={\left[\max\left(F_{1},F_{2}\right)\right]}^{n_{b}} \end{array}$$

where

(A31) $$\begin{array}{c} F_{1}=\frac{1-\rho /\rho_{0}}{1-\rho_{0}/\rho_{CJ}} \end{array}$$

(A32) $$\begin{array}{c} F_{2}=\{\begin{array}{cc} 0, & \;\mathrm{if}\;t\leq t_{b}\\ \left(t-t_{b}\right)/∆t_{b}, & \;\mathrm{if}\;t_{b}<t\leq t_{b}+∆t_{b}\\ 1, & \;\mathrm{otherwise} \end{array}\; \end{array}$$

where $n_{b}=2$ or 3, and $\rho_{CJ}$ is the density at the Chapman-Jouguet (C-J) state. The burn interval, $∆t_{b}$, is given as

(A33) $$\begin{array}{c} ∆t_{b}=m_{b}\frac{∆x}{V_{d}} \end{array}$$

where $m_{b}$ = 3–6 and $V_{d}$ is the detonation velocity.

The JWL EOS was used to model the thermodynamic state of the explosive gas, and can be expressed as

(A34) $$\begin{array}{c} p=\omega \rho e+A\left(1-\frac{\omega \theta }{R_{1}}\right)\exp\left(\frac{-R_{1}}{\theta }\right)+B\left(1-\frac{\omega \theta }{R_{2}}\right)\exp\left(\frac{-R_{2}}{\theta }\right) \end{array}$$

(A35) $$\begin{array}{c} e=\frac{p}{\rho \omega }-A\left(\frac{1}{\rho \omega }-\frac{1}{\rho_{0}R_{1}}\right)\exp\left(\frac{-R_{1}}{\theta }\right)-B\left(\frac{1}{\rho \omega }-\frac{1}{\rho_{0}R_{2}}\right)\exp\left(\frac{-R_{2}}{\theta }\right) \end{array}$$

(A36) $$\begin{array}{c} c^{2}=\frac{p\left(1+\omega \right)}{\rho }-A\left[\frac{\left(1+\omega \right)}{\rho }-\frac{R_{1}}{\rho \theta }\right]\exp\left(\frac{-R_{1}}{\theta }\right)-B\left[\frac{\left(1+\omega \right)}{\rho }-\frac{R_{2}}{\rho \theta }\right]\exp\left(\frac{-R_{2}}{\theta }\right) \end{array}$$

where $\theta =\rho /\rho_{0}$, $\rho_{0}$ is the reference density at room temperature, A and B. are the linear coefficients, $R_{1}$ and $R_{2}$ are the nonlinear coefficients, and ω is the unitless coefficient.

## Appendix B. Numerical Methods

### Appendix B.1. Third-Order Runge–Kutta Scheme

First, the governing equation (Equation (A37)) can be expressed as

(A37) $$\begin{array}{c} \frac{\partial U}{\partial t}=L\left(U\right) \end{array}$$

where

(A38) $$\begin{array}{c} L\left(U\right)=-\frac{\partial F}{\partial x}-\frac{\partial G}{\partial y}+S \end{array}$$

Applying the third-order Runge–Kutta scheme to Equation (A37) yields

(A39) $$\begin{array}{c} U^{\left(1\right)}=U^{n}+∆tL\left(U^{n}\right) \end{array}$$

(A40) $$\begin{array}{c} U^{\left(2\right)}=\frac{3}{4}U^{n}+\frac{1}{4}U^{\left(1\right)}+\frac{1}{4}∆tL\left(U^{\left(1\right)}\right) \end{array}$$

(A41) $$\begin{array}{c} U^{n+1}=\frac{1}{3}U^{n}+\frac{2}{3}U^{\left(2\right)}+\frac{2}{3}∆tL\left(U^{\left(2\right)}\right) \end{array}$$

### Appendix B.2. Third-Order CENO Scheme

The space and source terms are discretized using the numerical flux, ${\hat{f}}_{i\pm 1/2,j},\;{\hat{g}}_{i\pm 1/2,j}$, as

(A42) $$\begin{array}{c} L\left(U\right)=-\frac{1}{∆x}\left({\hat{f}}_{i+\frac{1}{2},j}-{\hat{f}}_{i-\frac{1}{2},j}\right)-\frac{1}{∆y}\left({\hat{g}}_{i,j+\frac{1}{2}}-{\hat{g}}_{i,j-\frac{1}{2}}\right)+S_{i,j} \end{array}$$

where the numerical fluxes are computed using the CENO scheme, and the source term, $S_{i,j}$, is obtained using the central difference method. The numerical flux is divided into two numerical fluxes as

(A43) $$\begin{array}{c} {\hat{f}}_{i+\frac{1}{2}}={\hat{f}}_{i+\frac{1}{2}}^{+}+{\hat{f}}_{i+\frac{1}{2}}^{-} \end{array}$$

The two numerical fluxes, ${\hat{f}}_{i+1/2}^{\pm }$, are computed using the third-order CENO scheme. The first-order CENO numerical flux is defined as

(A44) $$\begin{array}{c} {\hat{f}}_{i+\frac{1}{2}}^{+,1}=f_{i}^{+} \end{array}$$

where $f_{k}^{\pm }$ is the Lax–Friedrichs (LF) flux, given as

(A45) $$\begin{array}{c} f_{k}^{\pm }=\frac{1}{2}\left(f_{k}\pm \alpha u_{k}\right),\;k=i-2\;\ldots \;i+3 \end{array}$$

where

(A46) $$\begin{array}{c} \alpha =\{\begin{array}{c} CFL\cdot \frac{dx}{dt},\;\mathrm{for}\;\mathrm{global}\;\mathrm{LF}\\ \underset{i\leq k\leq i+1}{max}\left(\lambda_{k}\right),\;\mathrm{for}\;\mathrm{local}\;\mathrm{LF} \end{array} \end{array}$$

The second-order CENO numerical flux is computed as

(A47) $$\begin{array}{c} {\hat{f}}_{i+\frac{1}{2}}^{+,2}={\hat{f}}_{i+\frac{1}{2}}^{+,1}+\Psi \left(d_{1},d_{2}\right) \end{array}$$

where

(A48) $$\begin{array}{c} d_{1}={\hat{f}}_{i+\frac{1}{2}}^{+,2a}-{\hat{f}}_{i+\frac{1}{2}}^{+,1} \end{array}$$

(A49) $$\begin{array}{c} d_{2}={\hat{f}}_{i+\frac{1}{2}}^{+,2b}-{\hat{f}}_{i+\frac{1}{2}}^{+,1} \end{array}$$

(A50) $$\begin{array}{c} {\hat{f}}_{i+\frac{1}{2}}^{+,2a}=-\frac{1}{2}f_{i-1}+\frac{3}{2}f_{i} \end{array}$$

(A51) $$\begin{array}{c} {\hat{f}}_{i+\frac{1}{2}}^{+,2b}=\frac{1}{2}f_{i}+\frac{1}{2}f_{i+1} \end{array}$$

(A52) $$\begin{array}{c} \Psi \left(d_{1},d_{2}\right)=\{\begin{array}{c} \max\left[0,\min\left(\beta d_{1},d_{2}\right),\min\left(d_{1},\beta d_{2}\right)\right]\;\mathrm{if}\;d_{2}\geq 0\;\\ \min\left[0,\max\left(\beta d_{1},d_{2}\right),\max\left(d_{1},\beta d_{2}\right)\right]\;\mathrm{otherwise} \end{array} \end{array}$$

where 1≤β≤2. β=1 and β=2 represent the minmod limiter and the superbee limiter, respectively. Lastly, the third-order CENO numerical flux can be obtained using:

(A53) $$\begin{array}{c} {\hat{f}}_{i+\frac{1}{2}}^{+,3}={\hat{f}}_{i+\frac{1}{2}}^{+,2}+\mathrm{minmod}\left(d_{1},d_{2},d_{3}\right) \end{array}$$

where

(A54) $$\begin{array}{c} d_{1}={\hat{f}}_{i+\frac{1}{2}}^{+,3a}-{\hat{f}}_{i+\frac{1}{2}}^{+,2} \end{array}$$

(A55) $$\begin{array}{c} d_{2}={\hat{f}}_{i+\frac{1}{2}}^{+,3b}-{\hat{f}}_{i+\frac{1}{2}}^{+,2} \end{array}$$

(A56) $$\begin{array}{c} d_{3}={\hat{f}}_{i+\frac{1}{2}}^{+,3c}-{\hat{f}}_{i+\frac{1}{2}}^{+,2} \end{array}$$

(A57) $$\begin{array}{c} {\hat{f}}_{i+\frac{1}{2}}^{+,3a}=\frac{1}{3}f_{i-2}-\frac{7}{6}f_{i-1}+\frac{11}{6}f_{i} \end{array}$$

(A58) $$\begin{array}{c} {\hat{f}}_{i+\frac{1}{2}}^{+,3b}=-\frac{1}{6}f_{i-1}+\frac{5}{6}f_{i}+\frac{1}{3}f_{i+1} \end{array}$$

(A59) $$\begin{array}{c} {\hat{f}}_{i+\frac{1}{2}}^{+,3c}=\frac{1}{3}f_{i}+\frac{5}{6}f_{i+1}-\frac{1}{6}f_{i+2} \end{array}$$

where the minmod limiter is defined as

(A60) $$\begin{array}{c} \mathrm{minmod}\left(d_{1},d_{2},d_{3}\right)=\{\begin{array}{c} \min(d_{1},d_{2},d_{3})\;\mathrm{if}\;d_{i}>0\;\mathrm{for}\;\forall i\\ \max(d_{1},d_{2},d_{3})\;\mathrm{if}\;d_{i}<0\;\mathrm{for}\;\forall i\\ 0\;\mathrm{otherwise} \end{array} \end{array}$$

### Appendix B.3. Fifth-Order WENO Scheme

The fifth-order WENO numerical flux can be calculated using the weighted average of the first-, second-, and third-order numerical fluxes as

(A61) $$\begin{array}{c} {\hat{f}}_{i+\frac{1}{2}}^{+}=\omega_{1}{\hat{f}}_{i+\frac{1}{2}}^{+,\left(1\right)}+\omega_{2}{\hat{f}}_{i+\frac{1}{2}}^{+,\left(2\right)}+\omega_{3}{\hat{f}}_{i+\frac{1}{2}}^{+,\left(3\right)} \end{array}$$

where

(A62) $$\begin{array}{c} {\hat{f}}_{i+\frac{1}{2}}^{+,\left(1\right)}=\frac{1}{3}f_{i-2}-\frac{7}{6}f_{i-1}+\frac{11}{6}f_{i} \end{array}$$

(A63) $$\begin{array}{c} {\hat{f}}_{i+\frac{1}{2}}^{+,\left(2\right)}=-\frac{1}{6}f_{i-1}+\frac{5}{6}f_{i}+\frac{1}{3}f_{i+1} \end{array}$$

(A64) $$\begin{array}{c} {\hat{f}}_{i+\frac{1}{2}}^{+,\left(3\right)}=\frac{1}{3}f_{i}+\frac{5}{6}f_{i+1}-\frac{1}{6}f_{i+2} \end{array}$$

Here, the weighting factors are given as

(A65) $$\begin{array}{c} \omega_{k}=\frac{a_{k}}{\sum a_{k}} \end{array}$$

(A66) $$\begin{array}{c} a_{k}=\frac{\gamma_{k}}{{\left(\varepsilon +s_{k}\right)}^{2}} \end{array}$$

(A67) $$\begin{array}{c} \gamma_{1}=0.1 \end{array}$$

(A68) $$\begin{array}{c} \;\gamma_{2}=0.6 \end{array}$$

(A69) $$\begin{array}{c} \;\gamma_{3}=0.3 \end{array}$$

(A70) $$\begin{array}{c} s_{1}=\frac{13}{12}{\left(f_{i-2}-2f_{i-1}+f_{i}\right)}^{2}+\frac{1}{4}{\left(f_{i-2}-4f_{i-1}+3f_{i}\right)}^{2} \end{array}$$

(A71) $$\begin{array}{c} s_{2}=\frac{13}{12}{\left(f_{i-1}-2f_{i}+f_{i+1}\right)}^{2}+\frac{1}{4}{\left(f_{i-1}-f_{i+1}\right)}^{2} \end{array}$$

(A72) $$\begin{array}{c} s_{3}=\frac{13}{12}{\left(f_{i}-2f_{i+1}+f_{i+2}\right)}^{2}+\frac{1}{4}{\left(3f_{i}-4f_{i+1}+f_{i+2}\right)}^{2} \end{array}$$

where $\varepsilon \approx 10^{-6}$.

### Appendix B.4. Level-Set Method for Interface Tracking

If the velocity vector on the material interface is (u,v), the advection equation of the interface can be expressed as

(A73) $$\begin{array}{c} \frac{\partial \phi }{\partial t}+u\frac{\partial \phi }{\partial x}+v\frac{\partial \phi }{\partial y}=0 \end{array}$$

where ϕ is the signed distance function. Equation (A73) is solved using the third-order Runge–Kutta and the fifth-order WENO methods. While solving Equation (A73), ϕ may lose the signed distance property and may need to be re-initialized using the following equation:

(A74) $$\begin{array}{c} \frac{\partial \phi }{\partial \tau }+S\left(\phi \right)\left(\sqrt{\phi_{x}^{2}+\phi_{y}^{2}}-1\right)=0 \end{array}$$

where S(ϕ) can be expressed using the function proposed by Peng et al. [39] as

(A75) $$\begin{array}{c} S\left(\phi \right)=\frac{\phi }{\sqrt{\phi^{2}+\left(\phi_{x}^{2}+\phi_{y}^{2}\right){\left(∆x\right)}^{2}}} \end{array}$$

Subsequently, Equation (A74) can be discretized as

(A76) $$\begin{array}{c} \frac{\partial \phi }{\partial \tau }=L\left(\phi \right) \end{array}$$

(A77) $$\begin{array}{c} L\left(\phi \right)=-S^{+}\left(\sqrt{\max\left[{\left(a^{+}\right)}^{2},{\left(b^{-}\right)}^{2}\right]+\max\left[{\left(c^{+}\right)}^{2},{\left(d^{-}\right)}^{2}\right]}-1\right)\\ -S^{-}\left(\sqrt{\max\left[{\left(a^{-}\right)}^{2},{\left(b^{+}\right)}^{2}\right]+\max\left[{\left(c^{-}\right)}^{2},{\left(d^{+}\right)}^{2}\right]}-1\right) \end{array}$$

where

(A78) $$\begin{array}{c} a^{+}=\max\left(a,0\right) \end{array}$$

(A79) $$\begin{array}{c} \;a^{-}=\min\left(a,0\right) \end{array}$$

(A80) $$\begin{array}{c} a=\phi_{x}^{-} \end{array}$$

(A81) $$\begin{array}{c} \;b=\phi_{x}^{+} \end{array}$$

(A82) $$\begin{array}{c} \;c=\phi_{y}^{-} \end{array}$$

(A83) $$\begin{array}{c} d=\phi_{y}^{+} \end{array}$$

(A84) $$\begin{array}{c} \phi_{x}^{\pm },\;\phi_{y}^{\pm }\to \mathrm{WENO}5 \end{array}$$

The CFL condition is

(A85) $$\begin{array}{c} ∆\tau =CFL_{∆\tau }\cdot ∆x \end{array}$$

where $CFL_{∆\tau }\approx 0.3$.

## References

[1] Walters W.P., Zukas J.A. (1989). Fundamentals of Shaped Charges.

[2] Żochowski P., Warchoł R., Miszczak M., Nita M., Pankowski Z., Bajkowski M. (2021). Experimental and Numerical Study on the PG-7VM Warhead Performance against High-Hardness Armor Steel. Materials.

[3] Duprey K., Saucier E. Separation Systems Comparison for ARES I Launch Vehicle. Proceedings of the 44th AIAA/ASME/SAE/ASEE Joint Propulsion Conference & Exhibit.

[4] Molinari J.F. (2002). Finite element simulation of shaped charges. Finite Elem. Anal. Des..

[5] Liu M.B., Liu G.R., Lam K.Y., Zong Z. (2003). Meshfree particle simulation of the detonation process for high explosives in shaped charge unlined cavity configurations. Shock Waves.

[6] Liu M.B., Liu G.R., Zong Z., Lam K.Y. (2003). Computer simulation of high explosive explosion using smoothed particle hydrodynamics methodology. Comput. Fluids.

[7] Ma T., Wang C., Ning J. (2009). Numerical study on the shaped charges. Engineering Plasticity and Its Applications from Nanoscale to Macroscale: (with CD-ROM), Proceedings of the 9th AEPA2008, Daejeon, Korea, 20–24 October 2008.

[8] Kim K.H., Yoh J.J. (2008). Shock compression of condensed matter using multimaterial reactive ghost fluid method. J. Math. Phys..

[9] Zhang X., Wu C., Huang F. (2010). Penetration of shaped charge jets with tungsten–copper and copper liners at the same explosive-to-liner mass ratio into water. Shock Waves.

[10] Chen Q., Liu K. (2012). A high-resolution Eulerian method for numerical simulation of shaped charge jet including solid–fluid coexistence and interaction. Comput. Fluids.

[11] Sambasivan S.K., Udaykumar H.S. (2011). A sharp interface method for high-speed multi-material flows: Strong shocks and arbitrary materialpairs. Int. J. Comput. Fluid Dyn..

[12] Sambasivan S., Kapahi A., Udaykumar H.S. (2013). Simulation of high speed impact, penetration and fragmentation problems on locally refined Cartesian grids. J. Comput. Phys..

[13] Feng D.L., Liu M.B., Li H.Q., Liu G.R. (2013). Smoothed particle hydrodynamics modeling of linear shaped charge with jet formation and penetration effects. Comput. Fluids.

[14] Yi J., Wang Z., Yin J., Zhang Z. (2019). Simulation study on expansive jet formation characteristics of polymer liner. Materials.

[15] Pyka D., Kurzawa A., Bocian M., Bajkowski M., Magier M., Sliwinski J., Jamroziak K. (2020). Numerical and experimental studies of the ŁK type shaped charge. Appl. Sci..

[16] Du Y., He G., Liu Y., Guo Z., Qiao Z. (2021). Study on Penetration Performance of Rear Shaped Charge Warhead. Materials.

[17] Peng J., Jiang J., Men J., Li J., Zhou D., Hu Y. (2022). The Penetration–Explosion Effects of Differently Distributed Inactive/Active Composite Shaped Charge Jets. Materials.

[18] Liu X.D., Osher S. (1998). Convex ENO high order multi-dimensional schemes without field by field decomposition or staggered grids. J. Comput. Phys..

[19] Fedkiw R.P., Aslam T., Merriman B., Osher S. (1999). A non-oscillatory Eulerian approach to interfaces in multimaterial flows (the ghost fluid method). J. Comput. Phys..

[20] Johnson G.R., Cook W.H. (1985). Fracture characteristics of three metals subjected to various strains, strain rates, temperatures and pressures. Eng. Fract. Mech..

[21] Ponthot J.P. (1998). An extension of the radial return algorithm to account for rate-dependent effects in frictional contact and visco-plasticity. J. Mater. Process. Technol..

[22] Ponthot J.P. (2002). Unified stress update algorithms for the numerical simulation of large deformation elasto-plastic and elasto-viscoplastic processes. Int. J. Plast..

[23] Shu C.W. (2003). High-order finite difference and finite volume WENO schemes and discontinuous Galerkin methods for CFD. Int. J. Comput. Fluid Dyn..

[24] Sethian J.A. (1999). Level Set Methods and Fast Marching Methods: Evolving Interfaces in Computational Geometry, Fluid Mechanics, Computer Vision, and Materials Science.

[25] Udaykumar H.S., Tran L., Belk D.M., Vanden K.J. (2003). An Eulerian method for computation of multimaterial impact with ENO shock-capturing and sharp interfaces. J. Comput. Phys..

[26] Mehmandoust B., Pishevar A.R. (2009). An Eulerian particle level set method for compressible deforming solids with arbitrary EOS. Int. J. Numer. Methods Eng..

[27] Tran L., Udaykumar H.S. (2004). A particle-level set-based sharp interface cartesian grid method for impact, penetration, and void collapse. J. Comput. Phys..

[28] Camacho G.T., Ortiz M. (1997). Adaptive Lagrangian modelling of ballistic penetration of metallic targets. Comput. Methods Appl. Mech. Eng..

[29] Cooper S.R., Benson D.J., Nesterenko V.F. (2000). A numerical exploration of the role of void geometry on void collapse and hot spot formation in ductile materials. Int. J. Plast..

[30] Shimamura K., Ootsuka T. (2013). Study of water entry of high-špeed projectile. Procedia Eng..

[31] Saran S., Ayısıt O., Yavuz M.S. (2013). Experimental investigations on aluminum shaped charge liners. Procedia Eng..

[32] Sun S., Jiang J., Wang S., Men J., Li M., Wang Y. (2021). Comparison of Shaped Charge Jet Performance Generated by Machined and Additively Manufactured CuSn10 Liners. Materials.

[33] Rolc S., Buchar J., Akstein Z. Computer simulation of explosively formed projectiles (EFP). Proceedings of the 23rd International Symposium on Ballistics.

[34] Cheng X., Huang G., Liu C., Feng S. (2018). Design of a novel linear shaped charge and factors influencing its penetration performance. Appl. Sci..

[35] Soares G.C., Hokka M. (2021). The Taylor–Quinney coefficients and strain hardening of commercially pure titanium, iron, copper, and tin in high rate compression. Int. J. Impact Eng..

[36] Khan A.S., Huang S. (1995). Continuum Theory of Plasticity.

[37] Lemons D.S., Lund C.M. (1999). Thermodynamics of high temperature, Mie–Gruneisen solids. Am. J. Phys..

[38] Heuzé O. (2012). General form of the Mie–Grüneisen equation of state. Comptes Rendus Mec..

[39] Peng D., Merriman B., Osher S., Zhao H., Kang M. (1999). A PDE-based fast local level set method. J. Comput. Phys..

